# Stick to Convention or Bring Forth the New? Research on the Relationship Between Employee Conscientiousness and Job Crafting

**DOI:** 10.3389/fpsyg.2020.01038

**Published:** 2020-05-26

**Authors:** Xiayi Liu, Ting Yu, Wenhai Wan

**Affiliations:** ^1^Nanjing University, Nanjing, China; ^2^Huaqiao University, Quanzhou, China

**Keywords:** employee conscientiousness, error management climate, work promotion focus, work prevention focus, job crafting

## Abstract

Integrating regulatory focus theory and personality literature, we develop and test a moderated mediation model to specify the mediation mechanisms and boundary conditions of the association between employee conscientiousness and job crafting. Two-wave data collected from 389 employees and 95 supervisors showed that: Employee conscientiousness was positively associated with work promotion focus and work prevention focus. Employee conscientiousness was positively related to job crafting via work promotion focus, negatively related to job crafting via work prevention focus. Error management climate positively moderated the relationship between employee conscientiousness and work promotion focus, negatively moderated the relationship between employee conscientiousness and work prevention focus. The indirect relationship between employee conscientiousness and job crafting through work promotion focus was more pronounced under positive error management climate rather than negative, whereas the indirect relationship through work prevention focus was more pronounced under negative error management climate rather than positive.

## Introduction

Over the past two decades, extensive research on conscientiousness has suggested it to be valid predictor of performance ratings across jobs (Barrick and Mount, 1991; Dudley et al., 2006; Ohme and Zacher, 2015; Hassan et al., 2016). This is not surprising considering that conscientious individuals are not merely persistent, hardworking, and motivated in goal-directed behaviors, but also self-disciplined, careful, and morally scrupulous (Barrick and Mount, 1991; Costa et al., 1991). In line with this view, conscientiousness has previously been observed to have positive consequences on subjective well-being (Deneve and Cooper, 1998; Dyrenforth et al., 2010; Smith et al., 2013), health-related behaviors (Bogg and Roberts, 2004; Takahashi et al., 2013), relationship and job satisfaction (Judge et al., 2002; Lapierre and Hackett, 2011; Claxton et al., 2012). Yet, despite these positive connotations, much uncertainty still exists about the relationship between employee conscientiousness and job crafting behaviors (Bell and Njoli, 2016; Rudolph et al., 2017). Given the crucial role job crafting plays in facilitating organizational change successfully (Petrou et al., 2018), the primary purpose of this investigation is to unravel ***when and why*** conscientious employees are more likely to craft their jobs.

Job crafting is defined by Tims et al. (2013) as the “self-initiated change behaviors that employees engage in with the aim to align their jobs with their own preferences, motives, and passions” (P.173). This indicates that job crafting includes actions initiated by focal individuals to improve certain organizational processes or practices that they perceive to be dysfunctional for the organization (Wrzesniewski and Dutton, 2001; Bipp and Demerouti, 2014; Akkermans and Tims, 2017). In fact, the benefits of job crafting behaviors have previously been observed for both the individual and others in the same working team or even the whole organization (Akkermans and Tims, 2017; Bindl et al., 2019; Zhang and Parker, 2019). In this regard, conscientious employees should be more likely to craft their jobs, which can satisfy their needs for achievement and commitment to work (Simmering et al., 2003; Tims et al., 2013). Interestingly, however, job crafting is distinctive from other related initiative positive behaviors (for example, take charge behavior) in that job crafting is mainly oriented toward personal needs rather than improving others’ work or organizational performance (Akkermans and Tims, 2017; Kuijpers et al., 2020). From this viewpoint, job crafting behaviors may be unhelpful or even harmful for others or the organization since it may go beyond formal organizational goals or procedures (Campbell and Wiernik, 2015; Dierdorff and Jensen, 2018). As such, conscientious employees tend to avoid job crafting behaviors due to their internal moral scrupulousness and cautiousness (Tims et al., 2013; Bell and Njoli, 2016).

These inconsistent findings highlight the need to further explore the underlying psychological mechanisms and boundary conditions of the association between employee conscientiousness and job crafting behaviors. In this study, we propose this complex and paradoxical relationship to be aligned with different mediation mechanisms that either enhance or inhibit job crafting behaviors. To do so, we integrate relevant personality literature and regulatory focus theory (Higgins, 1997, 1998) as our overarching theoretical framework. This integration is appropriate because distal factors (personality traits) that are hardly to influence work behaviors directly can exert their effects via proximal motivational states (e.g., self-regulatory focus) (Chen et al., 2000; Wallace and Chen, 2006; Lanaj et al., 2012). By adopting this distal-proximal approach, we can formulate the underlying mechanisms to explain above inconsistent conclusions between employee conscientiousness and job crafting behaviors. Specifically, we expect that conscientiousness is likely to have opposite effects on job crafting behaviors by stimulating different work regulatory focus. In addition, more and more personality literature since the early 2000s has emphasized the importance of situation on the effects of any traits on work behaviors (Barrick and Mount, 2005; Rauthmann et al., 2015; Zaccaro et al., 2018). Regulatory focus theory also indicates that dispositional and contextual variables can jointly influence employee’s work-specific regulatory focus (Higgins, 1997, 2000; Forster et al., 2003). In conformity to these interactionist perspectives (personal and situational characteristics), the influence of employee conscientiousness may depend on situational features that activate or neutralize certain work regulatory focus. We propose error management climate (Van Dyck et al., 2005) as such one situational stimuli that fosters or inhibits the extent to which employee conscientiousness translates into job crafting behaviors via work promotion focus or work prevention focus.

Our research contributes to existing organizational literature in two major ways. First, we extend understandings regarding the previously unclear relationship between employee conscientiousness and job crafting behaviors. In contrast to prior findings that directly investigate the relationship between employee conscientiousness and job crafting behaviors, our study takes a systematic, distal-proximal view, suggesting that employee conscientiousness may not have a direct impact. By examining the potential mediatory mechanisms underlying this relationship, our research echo Rudolph et al. (2017) call to investigate the potential mechanisms between personality traits and job crafting behaviors. Our second theoretical contribution is in identifying the potential boundary conditions of conscientiousness by testing the moderating role error management climate plays. In doing so, we contribute to the literature by identifying situations where conscientious employees may adopt either a promotion focus strategy or a prevention focus strategy, which was positively or negatively associated with job crafting behaviors, respectively. Overall, this research should make an important contribution to the field of personality research by integrating the historically conflicting effects of employee conscientiousness on job crafting behaviors.

## Theoretical Development and Hypotheses

### Overview of Regulatory Focus Theory

To account for individual differences during goal pursuits, regulatory focus theory distinguishes two coexisting self-regulatory systems, namely promotion focus and prevention focus (Higgins, 1997, 2000). A promotion focus strategy is connected with nurturance needs, which directs people’ s attention toward advancement, aspiration, and accomplishment. This strategic means promotes people to accomplish tasks by approaching means that maximize gains and minimize non-gains. In contrast, a prevention focus strategy is relevant to vigilance needs or compliance with work-related regulations, which makes people more sensitive to negative consequences of organizational behaviors. This strategic means pulls people away from behaviors with potential negative performance implications. That is to say, employees performing a task can adopt either a promotion focus to maximize the positive outcomes, or a prevention focus to minimize the negative consequences (Higgins, 2000; Wallace and Chen, 2006). Nevertheless, the use of one regulatory focus might bring more benefits than the other in some situations. In most jobs, employees may face different situational requirements over time and therefore need to adopt promotion or prevention focus at different times appropriately to do their jobs better (Wallace and Chen, 2006). Taken together, employees’ work-specific regulatory focus can be crafted by a combination of personal and situational factors (Higgins, 1997, 2000; Forster et al., 2003; Lanaj et al., 2012). Next, we will use the regulatory focus theory as an overarching theoretical framework to develop our hypotheses.

### Employee Conscientiousness and Work Regulatory Focus

A conscientious person is believed to possess both proactive and inhibitive qualities. The proactive side comprises the volitional qualities such as the need for achievement and commitment to work; the inhibitive side encompasses qualities of dependability, thoroughness, moral scrupulousness, and cautiousness (Costa et al., 1991; Moon, 2001; Barrick et al., 2002). As such, conscientious employees tend to perform better due to their higher levels of achievement strivings, resulting in the formation of a promotion focus. They may simultaneously behave more safely as they have higher levels of cautiousness, resulting in the formation of a prevention focus. In other words, employee conscientiousness might lead to work promotion focus mainly through its proactive quality and that it also leads to prevention focus primarily through its inhibitive quality. Several empirical studies have already supported proactive quality and inhibitive quality (respectively) have a positive impact on promotion focus and prevention focus (Higgins and Spiegel, 2004; Wallace et al., 2009; Gorman et al., 2012; Costantini and Perugini, 2016).

Although a relatively small body of literature uses the specific dimensions of conscientiousness to examining their effects (Moon, 2001; Moon et al., 2008; Subrahmaniam et al., 2013; Chae et al., 2019) we choose to treat it as a unified construct given the two aspects of conscientiousness are expected to be highly and positively correlated with each other (Costa et al., 1991; Barrick et al., 2002). By doing so, we are able to simultaneously examine the potentially different indirect effects of employee conscientiousness on job crafting behaviors, hence possibly resolve the contradiction in prior studies. In this paper, we argue that conscientiousness appears to be positively related to both promotion focus and prevention focus.

H1a: Conscientiousness has a positive association with work promotion focus.
H1b: Conscientiousness has a positive association with work prevention focus.

### The Mediating Role of Work Regulatory Focus

Job crafting was defined as initiative and proactive change behaviors that employees make with the intention to fit better at work for themselves (Wrzesniewski and Dutton, 2001; Bruning and Campion, 2018). As contemporary work context changes so quickly, organizations increasingly need to rely on employees’ self-initiated, bottom-up efforts to identify and solve problems (Dierdorff and Jensen, 2018; Petrou et al., 2018). Prior studies have demonstrated that individuals who take actions to craft their jobs are likely to shape their jobs in ways in ways that fit better their interests, skills, and motivation (Wrzesniewski et al., 2010). In this regard, job crafting behaviors are beneficial for improving employees’ adjustment to organizational change (Petrou et al., 2018), mitigating the negative impacts value incongruence has on employees’ performance (Vogel et al., 2016), and promoting an individual’s engagement to work (Leana et al., 2009). However, a distinct characteristic of job crafting behaviors is that they are inherently oriented toward satisfaction of one’s own personal needs at work. To this point, job crafting behaviors may depart from the prescribed job tasks and work routines, thus having dysfunctional consequences for individual outcomes and/or the overall organization due to the misalignments between organizational goals and individual actions (Dierdorff and Jensen, 2018). To sum up, job crafting behaviors have the potential to positively or negatively impact organizational performance goals, especially when taking what is formally required in a given job into consideration.

According to regulatory focus theory, employees can adopt different work-specific regulatory focus to help with their goal achievement strategies (Higgins, 1998, 2000). Employees with a work promotion focus would like to pay more attention to achievement and task completion, striving to accomplish tasks by solving problems more quickly and easily (Tumasjan and Braun, 2012; Koopman et al., 2019). In terms of this view, adoption of a work promotion focus would sensitize employees to the potential positive consequences of job crafting behaviors, such as developing a more profound understanding of their work, exerting increased motivational effects on individuals, enhancing the capacity for emergent and complex problems, which would allow them to actualize their job more effectively (Higgins and Spiegel, 2004; Mäkikangas, 2018; Dubbelt et al., 2019). As such, employees with a promotion focus are more likely to have a stronger tendency to take actions to craft their jobs.

In contrast, adopting a work prevention focus may prompt employees to be more concerned about avoiding mistakes and negative outcomes (Higgins, 1998, 2000). They preferred to accomplish tasks within work values, beliefs and principles with a vigilance strategy (Higgins and Spiegel, 2004; Gamache et al., 2015; Koopman et al., 2019). Given the self-initiated nature of job crafting behaviors, employees who craft their jobs by themselves might go against formally documented job descriptions, which may include dysfunctional behaviors and therefore has potential negative consequences for organizational performance (Dierdorff and Jensen, 2018). Summering up the above analysis, it seems plausible to conclude that employees’ work prevention focus may negatively impact job crafting behaviors. As such, we hypothesize that:

H2a: Work promotion focus is positively associated with job crafting behaviors.
H2b: Work prevention focus is negatively associated with job crafting behaviors.

The core proposition of our study is the claim that work-specific regulatory focus may serve as a mediation mechanism to reconcile the contradictory relationship between employee conscientiousness and job crafting behaviors. According to Barrick and Mount (2005), researchers attempting to explore the effects of personality traits on work behaviors should investigate more proximal motivational factors in order to draw conclusions more properly. By integrating regulatory focus theory and personality literature, our research suggests that work-specific regulatory focus (motivational factors) can serve as such a role to mediate relations of employee conscientiousness (a distal individual personality predictor) with job crafting behavior (a specific work behavior) (Wallace and Chen, 2006; Lanaj et al., 2012; Niessen et al., 2016).

Given employee conscientiousness exerts positive impacts on work promotion focus and work prevention focus, which, in turn, show directionally opposite effects on job crafting behaviors, we expect the indirect effects of conscientiousness on job crafting to be positive or negative, depending on the mediator chosen. That is, different regulatory focuses serve as different proximal mechanisms underlying the distal relationship between employee conscientiousness and job crafting behaviors (Barrick and Mount, 2005; Lanaj et al., 2012). In consequence, it is plausible to suggest that employee conscientiousness has a positive association with work promotion, which in turn positively influences job crafting behaviors; employee conscientiousness positively related to work prevention focus, which in turn negatively impacts job crafting behaviors. As such, we hypothesize that:

H3a: Employee conscientiousness has a positive indirect effect on job crafting behaviors via work promotion focus.
H3b: Employee conscientiousness has a negative indirect effect on job crafting behaviors via work prevention focus.

### The Moderating Role of Error Management Climate

Error management climate can be defined as a set of shared norms, beliefs, and organizational practices concerning errors and mistakes management within an organization (Van Dyck et al., 2005). Prior studies have documented two types of error management climate, a positive one where errors are seen as opportunities for organizational learning enhancement versus a negative one in which individuals who make a mistake routinely get punished (Gronewold et al., 2013; Gold et al., 2014; Ameres et al., 2019). Specifically, a positive error management climate is characterized by acknowledging the inevitability of making errors, limiting sanctions for committing errors, exhibiting concern with correcting detecting errors, and encouraging employees to share error knowledge (Edmondson, 2004; Keith and Frese, 2011). In contrast, a negative error management climate is one where any errors may incur informal or even formal punishment, without recognizing the efforts employees make in attempting to better accomplish their tasks (Van Dyck et al., 2005; Keith and Frese, 2011; Frese and Keith, 2015). Since employees may adopt different work-specific regulatory focuses to fit situational requirement (Wallace and Chen, 2006), we argue that the strength of the mediating effects of work-specific regulatory focus depends on the error management climate.

According to regulatory focus theory, the adoption of a promotion focus strategy might increase safety incidents and errors at work due to the pursuit of getting work done more quickly (Higgins and Spiegel, 2004; Wallace and Chen, 2006). As stated above, the most prominent characteristic of a positive error management climate is the willingness to tolerate mistakes, we, therefore, argue that a positive error management climate may serve as an enhancing environmental factor activating employees’ achievement pursuit motivation, which in turn leading to work promotion focus. That is to say, conscientious employees are more likely to adopt a work promotion focus when exposed to a positive error management climate. Conversely, employees who adopt a work prevention focus strategy tend to value basic safety more (Higgins, 1998). Yet, a negative error management climate can be characterized as lacking tolerance for any error, where any errors may beget punishment (Van Dyck et al., 2005; Gronewold et al., 2013; Frese and Keith, 2015). Thus, it is logical to expect that conscientious employees under a negative error management climate will try to work safely due to a vigilant work focus. In other words, conscientious employees are more like to adopt a prevention focus when suffering a negative error management climate. Thus, it is hypothesized that:

H4a: Error management climate will positively moderate the relationship between employee conscientiousness and work promotion focus, such that this relationship becomes stronger when error management climate is positive (vs. negative).
H4b: Error management climate will negatively moderate the relationship between employee conscientiousness and work prevention focus, such that this relationship becomes stronger when error management climate is negative (vs. positive).

Work motivation researchers have proposed that proximal motivational processes can transmit the joint effects of stable personal attributes and malleable situational stimuli on work behaviors (Mitchell, 1997; Wallace and Chen, 2006). In line with this view, we propose that work-specific regulatory focus can serve as mediating mechanisms to explain how individual personality traits differently influence work behaviors according to differential conditions. The discussions above represent an integrated framework where different work-specific regulatory focus act as mediators between employee conscientiousness and job crafting behaviors, and error management climate conditions the effect of employee conscientiousness on work-specific regulatory focus. Based on this, we hypothesize that error management climate can further moderate the mediated relationship of employee conscientiousness, through work-specific regulatory focus on job crafting behaviors, that is, a moderated mediating effect. As such, we hypothesize that:

H5a: The indirect relationship between employee conscientiousness and job crafting behaviors through work promotion focus is positively moderated by error management climate, such that this indirect effect becomes stronger when error management climate is positive (vs. negative).
H5b: The indirect relationship between employee conscientiousness and job crafting behaviors through work prevention focus is negatively moderated by error management climate, such that this indirect effect becomes stronger when error management climate is negative (vs. positive).

## Method

### Participants and Procedure

To validate the theoretical framework, a two-wave (one-month interval) and multi-source (workgroup leaders and their direct reports) design has been adopted to minimize the potential influence of common source concerns (Podsakoff et al., 2003). Surveys were administered in 12 Chinese companies that represent diverse industries, including logistics, insurance, e-commerce, manufacturing, and software. With the assistance of internal coordinators (human resource personnel), we first made clear to participants the scientific research purpose only and the confidentiality of our survey. After completion, participants were instructed to return the survey directly to the researchers, in closed envelopes.

At time one, 534 employees rated their demographic characteristics, conscientiousness and error management climate. 459 employees returned completed surveys, representing a response rate of 85.96%. At time two, the 534 employees were invited to fill in a follow-up questionnaire assessing their work promotion focus and work prevention focus. And in the meantime, 108 leaders of the 534 employees rated followers’ levels of job crafting behaviors. 424 employees (79.40%) and 98 leaders (90.74%) completed the second survey. The final matched samples included 95 workgroup leaders and their 389 direct subordinates, yielding a response rate of 72.85% for employees and 87.96% for leaders.

The average team size was 4.09 members (with around 3 or 8members per team, and a standard deviation of SD = 1.13). The employee sample comprised of 176 men and 213 women. Age was coded into four categories (along with the percentage of sample in each category): below 20 years (1.3%), 21 to 30 years (52.7%), 31 to 40 years (36.0%), 41 to 50 years (8.7%), over 51 years (1.3%). Tenure was reported with for four bands: less than 3 years (67.1%), 4 to 6 years (15.4%), 7 to 9 years (7.7%), over 10 years (9.8%). In terms of education, 12.9% had a high school diploma or lower, 26.7% had completed a college degree, 40.1% held a bachelor degree, 20.3% had postgraduate qualifications or higher.

### Measures

We used a response format of 5-point scale (1 = strongly disagree to 5 = strongly agree, unless otherwise noted). To ensure all items can be clearly understood by every participant, we translate the English scales into Chinese following a back-translation procedure (Brislin, 1980).

#### Employee Conscientiousness

At time 1, employees completed a 12-item measure of their trait-level conscientiousness (Costa and McCrae, 1992). A sample item includes “When I make a commitment, I can always be counted on to follow through.” The coefficient α in this study was 0.956.

#### Error Management Climate

At time 1, employees rated their perceived error management climate using 16 items developed by Cigularov et al. (2010). A sample item reads “Although we make mistakes, we don’t let go of the final goal.” The coefficient α in this study was 0.948. Because error management climate is modeled as a group-level construct, we took three steps to justify the viability of aggregation by examining interrater reliability coefficient (*r*_wg_), intra-class correlation *ICC* (1) and reliability of group *ICC* (2) in sequence (Bliese, 1998). We obtain adequate value to support aggregation given the value of *r*_wg_ (0.966), *ICC* (1) (0.533) and *ICC* (2) (0.948) (James et al., 1993; LeBreton and Senter, 2008).

#### Work Regulatory Focus

At time 2, employees provided ratings of their work-specific regulatory focus with a 12-item measure from Wallace et al. (2009). This scale consists of two factors, namely work promotion focus and work prevention focus, each with six items. A sample item of work promotion focus reads “I focus on work activities that allow me to get ahead at work.” The coefficient α in this study was 0.886. A sample item of work prevention focus reads “I focus on completing work tasks correctly.” The coefficient α in this study was 0.887.

#### Job Crafting

At time 2, leaders rated their followers’ job crafting behaviors with a 4-item scale adapted from Leana et al. (2009): “This employee introduces new approaches to improve his/her work,” “This employee changes minor work procedures that he/she thinks are not productive,” “This employee changes the way he/she does his/her job to make it easier to himself/herself,” “This employee rearranges the position of relevant equipment in the workplace.” The coefficient α in this study was 0.871.

#### Control Variables

I controlled for several potential confounding variables at the individual and group level of analysis. Specifically, we considered employee gender, age, educational level and organizational tenure as individual level control variables, as well as team size as group level control variables.

### Analytic Strategy

Due to the nested data structure in our study, we adopted the multilevel path analysis with Mplus software (version 7.4) (Muthen and Muthen, 2010) to test the hypotheses addressed above. This approach allowed researchers to test all the relationships in our model simultaneously and to integrate tests of mediation and moderation using a bootstrapping methodology (Edwards and Lambert, 2007; Heck and Thomas, 2015). Specifically, we took four steps to test the hypotheses. Firstly, we estimated Level 1 direct effects of employee conscientiousness on work promotion focus and work prevention focus as well as the direct effects of work promotion focus and work prevention focus on job crafting behaviors to test hypotheses H1(a), H1(b), H2(a), H2(b). Then, we examined the mediation effect of H3(a), H3(b) by calculating 95% confidence intervals (CIs) of the bootstrap simulation (5000 iterations) as it has the advantage of bias correction (MacKinnon et al., 2004). Thirdly, to test the cross-level moderating effects of error management climate on (a) work promotion focus and (b) work prevention focus, level 1 predictor, employee conscientiousness, was group-mean centered and level 2 moderator, error management climate, was grand-mean centered. Lastly, we estimated the conditional indirect effects with the bootstrapping approach (using 5000 iterations). According to the recommendations by Preacher et al. (2010) and recent empirical multilevel moderated mediation research (e.g., Shi et al., 2013; Hu et al., 2018), 95% confidence intervals were used to verify the significance of hypothesized conditional indirect effects about H5(a) and H5(b).

## Results

### Confirmatory Factor Analyses

Prior to testing the proposed model in our study, we first carried out confirmatory factor analyses (CFA) by using individual-level data to assess whether the measures used in our study, namely employee conscientiousness, error management climate, work promotion focus, work prevention focus and job crafting have eligible discriminant validity. Based on the methods recommended by Anderson and Gerbing (1988), we tested the Chi-square differences between our five-factor baseline model and five alternative models to see which model mostly fit the data. The results in Table 1 suggested that our five-factor model provided a significantly better fit than the other five models (*χ^2^* = 2045.495, *df* = 892, *χ^2^/df* = 2.29, *CFI* = 0.904, *TLI* = 0.898, *RMSEA* = 0.058) (Steiger, 1990; Kline, 2015). As such, the results support the distinctiveness of key variables, enhancing our confidence in testing following hypotheses.

**TABLE 1:** Results of the confirmatory factor analyses of study variables.

Model	*χ^2^*	*df*	*χ^2^/df*	*CFI*	*TLI*	*RMSEA*	*Δχ2(Δdf)*^*a*^
1. 5-factor	2045.495	892	2.29	0.904	0.898	0.058	–
2. 4-factor	3245.778	896	3.62	0.804	0.793	0.082	1200.283 (4)
3. 4-factor	5351.609	896	5.97	0.627	0.607	0.113	3306.114 (4)
4. 3-factor	4231.140	899	4.71	0.721	0.707	0.098	2185.645 (7)
5. 2-factor	7131.635	901	7.92	0.479	0.453	0.133	5086.140 (9)
6. 1-factor	7777.177	902	8.62	0.425	0.397	0.140	5731.682 (10)

**N* = 389 individuals, 95 work groups. Model 1 (5-factor model) treats all five variables (conscientiousness, error management climate, work promotion focus, work prevention focus, and job crafting) as independent factors. Model 2 (4-factor model) combines two mediation variables (work promotion focus and work prevention focus) as one factor. Model 3 (4-factor model) combines the independent variable (conscientiousness) and moderate variable (error management climate) as one factor. Model 4 (3-factor model) combines the independent variable (conscientiousness) and mediation variables (work promotion focus and work prevention focus) as one factor. Model 5 (2-factor model) treats the dependent variable (job crafting) as one factor and combines the other four variables as one factor. Model 6 (1-factor model) combines all five variables as one factor. ^*a*^Models are compared with the five-factor baseline model.*

### Descriptive Statistics

Table 2 provided the descriptive statistics, reliabilities and correlations of the variables in our study. Consistent with our hypotheses, conscientiousness was positively related to both work promotion focus (*r* = 0.380, *p*<0.001) and work prevention focus(*r* = 0.236, *p*<0.01). In addition, work promotion focus has a positive correlation with job crafting (*r* = 0.322, *p*<0.001) while work prevention focus negatively correlated with job crafting (*r* = −0.297, *p*<0.001).

**TABLE 2:** Descriptive statistics and correlations for study variables.

Variables	*Mean*	*SD*	1	2	3	4	5	6	7	8
**Individual level**										
1. Gender^*a*^	0.45	0.50	–							
2. Age	2.56	0.72	0.088	–						
3. Education	2.68	0.94	−0.156**	−0.261***	–					
4. Tenure	1.60	0.99	−0.062	−0.105*	0.214***	–				
5. EC	3.45	0.86	0.036	−0.000	−0.044	−0.069	–			
6. PROF	2.94	0.86	0.090	0.063	−0.086	−0.001	0.380***	–		
7. PREF	3.01	0.87	0.009	0.090	−0.044	0.024	0.236***	−0.094	–	
8. JC	3.15	0.84	0.030	0.049	−0.005	−0.068	−0.007	0.322***	−0.297***	–
**Group level**										
9. Team size	4.09	1.13								
10. EMC	3.31	0.52								0.029

*N* = 389 individuals, 95 work groups. *p<0.05,**p<0.01, ***p<0.001. EC, Employee conscientiousness; PROF, Work promotion focus; PREF, Work prevention focus; JC, Job crafting; EMC, Error management climate. ^*a*^Employee gender was coded as 0 = female, 1 = male.

### Hypotheses Testing

Since the control variables in our sample were not significantly related to job crafting behaviors (see Table 2), for the sake of parsimony, we conducted subsequent analyses without the control variables.

**FIGURE 1:**
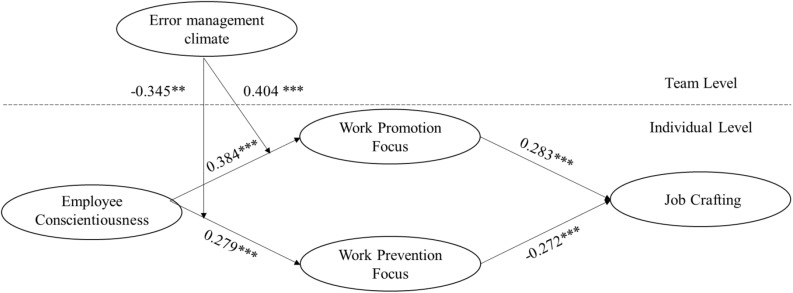
Path coefficients from the selected model.

The results presented in Supplementary Materials reveal that including these control variables does not affect the outcome of our Hypotheses tests, nor the interpretation of our findings. All hypothesized relationships were well supported by the results shown in Figure 1.

H1a and H1b proposed that employee conscientiousness has a positive association with both work promotion focus and work prevention focus. Figure 1 indicates that conscientiousness is positively related to both work promotion focus (*γ* = 0.384, *p*<0.001) and work prevention focus (*γ* = 0.279, *p*<0.001). Hence, H1a and H1b were well supported.

H2a and H2b hypothesized a positive relationship between work promotion focus and job crafting while a negative relationship between work prevention focus and job crafting. As shown in Figure 1, work promotion focus and work prevention focus were significantly related to employee job crafting (*γ* = 0.283, *p*<0.001, and *γ* = −0.272, *p*<0.001, respectively). Thus, H2a and H2b were well supported.

H3a predicted that employee conscientiousness has a positive and indirect effect on job crafting via work promotion focus, while H3b stated a negative and indirect effect via work prevention focus. The bootstrap simulation results demonstrated that there was a positive and indirect relationship between employee conscientiousness and job crafting behavior via work promotion focus (indirect effect = 0.109, 95% CI [0.054, 0.163]), a negative and indirect relationship between employee conscientiousness and job crafting behavior via work prevention focus (indirect effect = −0.076, 95% CI [−0.127, −0.024]). Hence, H3a and H3b received support.

**FIGURE 2:**
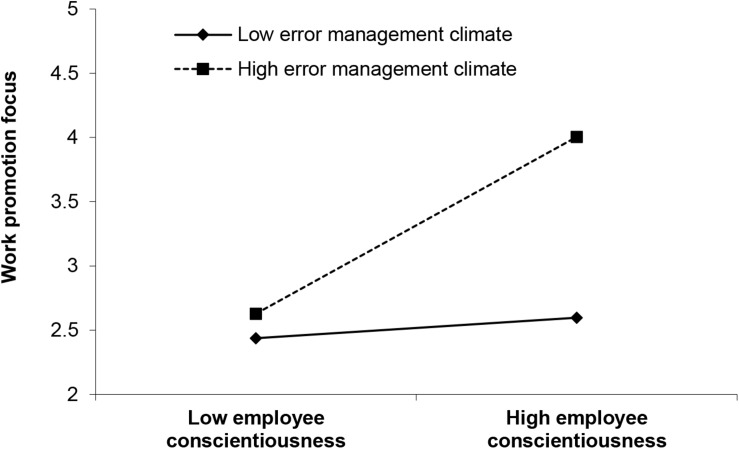
Interaction of error management climate and employee conscientiousness predicting work promotion focus.

**FIGURE 3:**
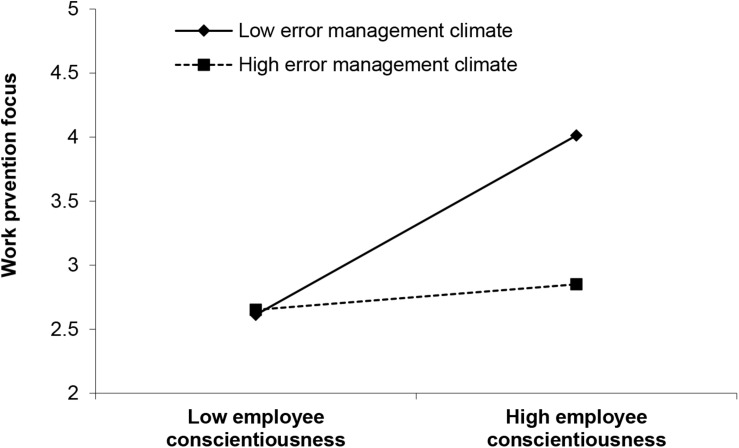
Interaction of error management climate and employee conscientiousness predicting work prevention focus.

H4a and H4b stated that error management climate moderates the relationship between employee conscientiousness and work promotion/prevention focus. As presented in Figure 1, error management climate positively and significantly moderates the relationship between employee conscientiousness and work promotion focus (*γ* = 0.404, *p*<0.001), but negatively and significantly moderates the relationship between employee conscientiousness and work prevention focus (*γ* = −0.345, *p*<0.01). We plotted the interactions to better illustrate the moderation effects (as shown in Figures 2, 3) (Aiken and West, 1991). In Figure 2, simple slope analyses suggested that there was a positive relationship between employee conscientiousness and work promotion focus when error management climate was high (1 SD above the mean; simple slope = 0.594, *t* = 4.318, *p*<0.001) but a nonsignificant relationship when error management climate was low (1 SD below the mean; simple slope = 0.174, *t* = 1.264, *ns*). As such, H4a was supported. In Figure 3, simple slope analyses revealed that employee conscientiousness and work prevention focus was positively related when error management climate was low (1 SD below the mean; simple slope = 0.458, *t* = 4.928, *p*<0.001) but was positively yet nonsignificantly related when error management climate was high (1 SD above the mean; simple slope = 0.100, *t* = 1.071, *ns*), supporting H4b.

H5a and H5b proposed that error management climate would moderate the indirect effects of employee conscientiousness on job crafting through (a) work promotion focus and (b) work prevention focus. The results in Table 3 showed that the conditional indirect effect of employee conscientiousness on job crafting through work promotion focus was stronger and significant under high error management climate (Effect size = 0.168, SE = 0.043, 95% CI [0.084, 0.253]) but was weaker under low error management climate (Effect size = 0.049, SE = 0.024, 95% CI [0.002, 0.096]). The significant difference between above two conditional indirect effects (Effect size = 0.119, SE = 0.043, 95% CI [0.036, 0.202]) indicated that error management climate serves as a critical role in conditioning the positive and indirect effect of employee conscientiousness on job crafting via work promotion focus. Similarly, the conditional indirect effect of employee conscientiousness on job crafting through work prevention focus was stronger and significant under low error management climate (Effect size = −0.125, SE = 0.036, 95% CI [−0.196, −0.054]) but was weaker and not significant under high error management climate (Effect size = −0.027, SE = 0.030,95% CI [−0.085, 0.031]). The significant difference between above two conditional indirect effects (Effect size = 0.098, SE = 0.040, 95% CI [0.019, 0.177]) suggested that error management climate serves as a critical role in conditioning the positive and indirect effect of employee conscientiousness on job crafting via work prevention focus. Thus, we have enough evidence to support H5a and H5b.

**TABLE 3:** Results for the conditional indirect effect of Employee Conscientiousness on Job Crafting via Work Promotion/ Prevention Focus across levels of error management climate.

Moderator	Effect size	Boot SE	LL 95% CI	UL 95% CI
error management climate	Employee Conscientiousness → Work Promotion Focus → Job Crafting			
High (+1 SD)	0.168	0.043	0.084	0.253
Low (−1 SD)	0.049	0.024	0.002	0.096
Difference	0.119	0.043	0.036	0.202
error management climate	Employee Conscientiousness → Work Prevention Focus → Job Crafting			
High (+1 SD)	−0.027	0.030	−0.085	0.031
Low (−1 SD)	−0.125	0.036	−0.196	−0.054
Difference	0.098	0.040	0.019	0.177

## Discussion

By integrating regulatory focus theory and relevant personality literature, this study attempts to carry out a deep exploration of the controversial relationship between employee conscientiousness and job crafting behaviors. By testing a multilevel, moderated mediation model with two-wave multisource data, we concluded that employee conscientiousness has a positive indirect effect on job crafting via work promotion focus, a negative indirect effect via work prevention focus, confirming the mediating role work-specific regulatory focus plays. In addition, error management climate was found to condition the mediating effect of work-specific regulatory focus. The results showed that conscientious employees under positive error management climate are more likely to possess work promotion focus and, subsequently, exhibit more job crafting behaviors. Conversely, the employee conscientiousness-job crafting relationship is much stronger when error management climate is negative and subsequently, exhibits fewer job crafting behaviors. As we describe below, this new understanding from this study should have several interesting theoretical and managerial implications.

### Theoretical Implications

Firstly, our findings contribute to personality literature by integrating a theoretical framework to clearly explain when and why conscientious employees might craft their jobs. Although previous studies have linked employee conscientiousness with job crafting behaviors (Simmering et al., 2003; Dahling et al., 2012; Tims et al., 2013; Bell and Njoli, 2016), there remains a paucity of a strong theoretical framework for addressing the debate in the personality literature concerning the impact of employee conscientiousness on job crafting behaviors. Accordingly, this paper integrating regulatory focus theory and relevant personality literature as an overarching framework, highlighting both the boundary conditions (error management climate) and mediation mechanisms (work promotion focus and work prevention focus) that drive the relationship. As such, our study echoes the call to build and test theory regarding the mediation mechanisms and boundary conditions to systematically explain the distal relationship between individual personality traits and specific behaviors (Barrick and Mount, 2005; Rudolph et al., 2017).

Secondly, the present study should make contributions to the literature of situational variation in trait expression. To date, several studies have indicated that situation serves an important role in conditioning the trait’s effect on behavior (Barrick and Mount, 2005; Funder, 2006; Zaccaro et al., 2018; Wood et al., 2019). Our study addresses this call by examining error management climate as a situational moderator of employee conscientiousness. Specifically, the indirect effects of employee conscientiousness on job crafting via work promotion/prevention focus were conditioned by error management climate. This finding is beneficial for future research to invest how personality traits relate to work behavior by incorporating error management climate as such one contextual factor into their study.

Finally, we shed light on research of the influences of conscientiousness. Currently, scholars often took a “too-much-of-a-good-thing effect” (TMGT) perspective, which suggests that “all seemingly positive relations reach context-specific inflection points after which the relations turn asymptotic and often negative, resulting in an overall pattern of curvilinearity” (Pierce and Aguinis, 2013; p. 313), to explain the complex relationship between employee conscientiousness and various workplace outcomes, such as job satisfaction, organizational citizenship and well-being (LaHuis et al., 2005; Le et al., 2011; Pierce and Aguinis, 2013). Yet, a recent study undertaken by Nickel et al. (2019) suggested that no evidence was found for systematic curvilinear associations between employee conscientiousness and the aforementioned outcomes. In this regard, we answer the call to explore how situational variables affect the relationship between employee conscientiousness and subsequent behaviors (Wallace and Chen, 2006).

### Practical Implications

The findings of our study also yield a number of important practical implications. Our work sheds new light on the complex relationship between employee conscientiousness and job crafting behaviors by highlighting the opposite mediating effects of work promotion focus and work prevention focus. The findings suggest that it is insufficient to ensure employees’ positive reactions to change only by improving their conscientiousness level. Instead, organizations should pay more attention to activate employees’ motivational states (for example, work promotion focus) because they directly influence employees’ observable job crafting behaviors. Therefore, organizations aimed at prompting employees’ certain proactive behavior should take a comprehensive consideration of both personality traits and inspiring approaches, which are all important in determining employees’ behaviors.

Our findings also indicate the critical role error management climate plays in strengthening the proximal motivational mechanism of conscientious employees’ work regulatory focus. Namely, even if two employees share a similar level of conscientiousness, they may behave in the opposite way due to the differences in working climates. Hence, organizations seeking to recruit employees with certain personality traits, such as conscientiousness, should also take work arrangement and work environment into consideration. Only by such consideration can organizations maximize the benefit as well as attenuate potential downsides of certain personality traits.

### Limitations and Future Research

Despite these promising results, our study also has several limitations that should be recognized. First, we relied on self-reports to assess conscientiousness, work promotion focus and work prevention focus, raising potential common method variance (CMV) bias (Podsakoff et al., 2012). This concern, however, is attenuated as we measured conscientiousness, work promotion focus and work prevention focus at different times. Additionally, where inferred, the relationships we proposed in our study have solid theoretical and empirical basis. In future investigation, however, we encourage researchers to utilize longitudinal research or experimental design to strengthen the causal claims.

Second, although our findings have partially opened the black box of the processes linking employee conscientiousness to job crafting, we encourage future researchers to take a further step in investigating the potential negative consequences of job crafting behaviors as well as the boundary conditions of these influences. Previous studies have established that the consequences of job crafting behaviors can be functional or dysfunctional (Wrzesniewski and Dutton, 2001; Petrou et al., 2018). In this regard, future research should be undertaken to explicate the changes employees make across the full spectrum of job crafting.

Third, our research focused on the moderating role of error management climate rather than other individual level variables. Previous studies have shown that personality trait combinations play a critical role in influencing work-related attitudes and outcomes (Witt, 2002; Perry et al., 2010). Therefore, future studies are recommended to combine conscientiousness with other personality traits to better understand the influence of employee conscientiousness on job crafting and other work-related outcomes.

## Data Availability Statement

The raw data supporting the conclusions of this article will be made available by the authors, without undue reservation, to any qualified researcher.

## Author Contributions

All authors listed have made a substantial, direct and intellectual contribution to the work, and approved it for publication.

## Conflict of Interest

The authors declare that the research was conducted in the absence of any commercial or financial relationships that could be construed as a potential conflict of interest.
