# Supplementary material for: Stick to Convention or Bring Forth the New? Research on the Relationship Between Employee Conscientiousness and Job Crafting
Source: Front Psychol. 2020 May 26;11:1038. doi: 10.3389/fpsyg.2020.01038 (PMC7265213; doi:10.3389/fpsyg.2020.01038)
Supplement: Supplementary file 1 [file Table_1.DOCX]

TITLE: A two-level first-stage moderated mediation path analysis model

DATA: FILE IS JCC.dat;

VARIABLE:

NAMES ARE ID X W PRO PRE Y; ! X=employee conscientiousness W=error management climate pro=work promotion focus PRE= work prevention focus Y=job crafting

USEVARIABLES ARE ID X W PRO PRE Y;

Cluster =ID;

WITHIN = X;

BETWEEN =W;

DEFINE:

CENTER W (GRANDMEAN); ! 水平-2 调节变量 w 总中心化

CENTER X (GROUPMEAN); ! 水平-1 预测变量 x 组中心化

ANALYSIS: TYPE = TWOLEVEL RANDOM;

MODEL:

%WITHIN%

s1 | PRO on X;

s2 | PRE on X;

Y on PRO (b1);

Y on PRE (b2);

%BETWEEN%

s1 on W (al); ! 跨层交互作用

[s1] (a0); ! 随机斜率均值

s2 on W (a3); ! 跨层交互作用

[s2] (a2); ! 随机斜率均值

PRO on W;

PRE on W;

PRO with s1;

PRE with s2;

Y with PRO;

Y with PRE;

Y with s1;

Y with s2;

Y with W;

MODEL CONSTRAINT:

NEW (ind1 ind2 ind_h1 ind_l1 diff1 ind_h2 ind_l2 diff2);

ind1=a0*b1;

ind2=a2*b2;

ind_h1 = (a0 + al*(.52))*b1; ! w 均值为0，SD 为 .52

ind_l1 = (a0 + al*(-.52))*b1;

Diff1 = ind_h1 - ind_l1; ! 显著表明有调节的中介效应存在

ind_h2 = (a2 + a3*(.52))*b2; ! w 均值为0，SD 为 .52

ind_l2 = (a2 + a3*(-.52))*b2;

Diff2 = ind_h2 - ind_l2; ! 显著表明有调节的中介效应存在

OUTPUT: SAMPSTAT CINTERVAL TECH1;
