# Supplementary material for: Stick to Convention or Bring Forth the New? Research on the Relationship Between Employee Conscientiousness and Job Crafting
Source: Front Psychol. 2020 May 26;11:1038. doi: 10.3389/fpsyg.2020.01038 (PMC7265213; doi:10.3389/fpsyg.2020.01038)
Supplement: Supplementary file 2 [file Table_2.DOCX]

# SUPPLEMENTAL MATERIALS

**
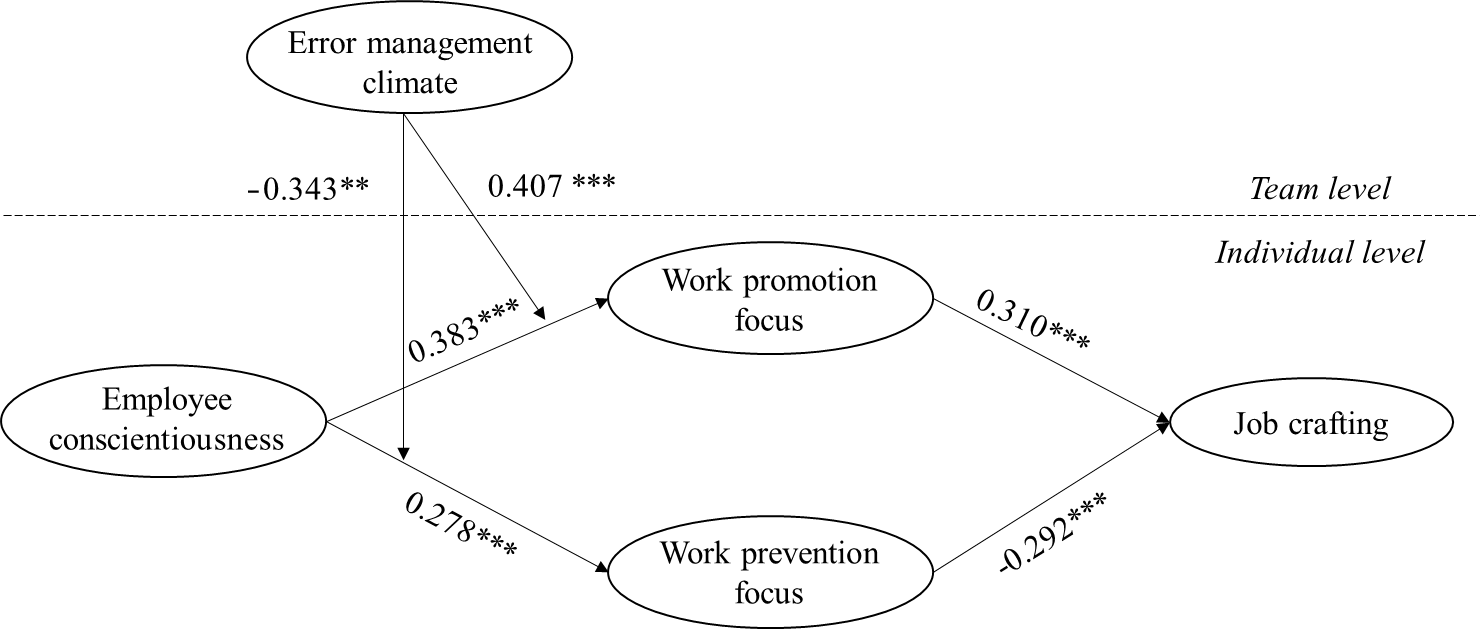
**Following established recommendations for statistical control (Becker et al., 2016; Hussain, Shu, Tangirala, & Ekkirala, 2019), we ran sensitivity analyses (i.e., running different types of regressions including and excluding certain variables; McEvoy & Cascio, 1987) to test the robustness of our results including the control variables. The results show that all the significant findings remained the same. Specifically, employee conscientiousness was positively related to work promotion focus (γ = 0.383, p < 0.001) and work prevention focus (γ = 0.278, p < 0.001). Work promotion focus has a positive association with job crafting (γ = 0.310, p < 0.001), while work prevention focus has a negative association with job crafting (γ = -0.292, p < 0.001). There was a positive indirect relationship between employee conscientiousness and job crafting behavior via work promotion focus (indirect effect = 0.127, 95% CI [0.078, 0.186]), a negative indirect relationship between employee conscientiousness and job crafting behavior via work prevention focus (indirect effect = -0.073, 95 % CI [-0.125, -0.034]). The interaction term of employee conscientiousness and error management climate was also significantly related to work promotion focus (γ = 0.407, p < 0.001) and work prevention focus (γ = -0.343, p < 0.001). In addition, the conditional indirect effect of employee conscientiousness on job crafting through work promotion focus was stronger and significant at high error management climate(Effect size = 0.184, SE=0.043，95% CI [0.099，0.269]) but was weaker at low error management climate(Effect size = 0.053, SE=0.025，95% CI [0.004，0.102]) .Similarly, the conditional indirect effect of employee conscientiousness on job crafting through work prevention focus was stronger and significant at low error management climate(Effect size = -0.131, SE=0.036，95% CI [-0.202，-0.060]) but was weaker and not significant at high error management climate(Effect size = -0.031, SE=0.030，95% CI [-0.090，0.028]).

Becker, T. E., Atinc, G., Breaugh, J. A., Carlson, K. D., Edwards, J. R., & Spector, P. E. (2016). Statistical control in correlational studies: 10 essential recommendations for organizational researchers. *Journal of Organizational Behavior, 37*(2), 157-167.

Hussain, I., Shu, R., Tangirala, S., & Ekkirala, S. (2019). The voice bystander effect: How information redundancy inhibits employee voice. *Academy of Management Journal, 62*(3), 828-849.

McEvoy, G. M., & Cascio, W. F. (1987). Do good or poor performers leave? A meta-analysis of the relationship between performance and turnover. *Academy of Management Journal, 30*(4), 744-762.
