# Supplementary material for: Stick to Convention or Bring Forth the New? Research on the Relationship Between Employee Conscientiousness and Job Crafting
Source: Front Psychol. 2020 May 26;11:1038. doi: 10.3389/fpsyg.2020.01038 (PMC7265213; doi:10.3389/fpsyg.2020.01038)
Supplement: Supplementary file 3 [file Table_3.DOCX]

Metadata of Raw Data

| **Variable** | **Description** |
| --- | --- |
| ID | Sanmples with the same ID work on the same team. |
| teamnum | “teamnum” means “teamnumber”, which captures the number of people in a team. |
| gender | 0=female, 1=male. |
| age | 1=below 20 years; 2=21 to 30 years; 3=31 to 40 years; 4=41 to 50 years; 5=over 51 years |
| edu | 1=high school diploma or lower; 2=college degree; 3=bachelor degree; 4=postgraduate qualifications or higher |
| time | 1=less than 3 years; 2=4 to 6 years; 3=7 to 9 years; 4=over 10 years |
| EC1-EC12 | EC1-EC12 capture Employee Conscientiousness |
| EMC1-EMC16 | EMC1-EMC16 capture Error Management Climate |
| RF1- RF6 | RF1- RF6 capture Work Promotion Focus |
| RF7- RF12 | RF7- RF12 capture Work Prevention Focus |
| JC1- JC4 | JC1- JC4 capture Job Crafting |
| industry | “industry” describes the industry the samples work in. |
